# Polydiacetylene-Based High-Throughput Screen for Surfactin Producing Strains of *Bacillus subtilis*

**DOI:** 10.1371/journal.pone.0088207

**Published:** 2014-02-03

**Authors:** Lingyan Zhu, Qing Xu, Ling Jiang, He Huang, Shuang Li

**Affiliations:** 1 College of Biotechnology and Pharmaceutical Engineering, Nanjing University of Technology, Nanjing, People's Republic of China; 2 College of Food Science and Light Industry, Nanjing University of Technology, Nanjing, People's Republic of China; University of Groningen, Groningen Institute for Biomolecular Sciences and Biotechnology, Netherlands

## Abstract

Although traditional mutation is still an attractive approach for strain improvement, it is tedious, time-consuming, and inefficient to screen for surfactin producing strains. To overcome this, we developed a high-throughput screening method for surfactin producing mutants by applying polydiacetylene (PDA) vesicles as sensors with visible chromatic change from blue to red, detected as colorimetric response (CR%) signal, which can even semi-quantify the yields of surfactin. *Bacillus subtilis* 723 was used as parent strain and multiply mutated with atmospheric and room temperature plasma (ARTP). Mutants were cultured in MicroFlask by Duetz (24 square deepwell plates, Applikon Biotechnology) and surfactin titers were tested in 96-well plates with PDA vesicles. Mutants with surfactin titers above150 mg/L (CR% value above 26%) were selected as high-yield strains and further quantified by HPLC. By integrating MicroFlask cultivation and the PDA vesicles detection, we screened 27,000 mutants and found 37 high-yield strains. From these, one mutant produced 473.6 mg/L surfactin (including 353.1 mg/L C_15_ surfactin), which was 5.4-fold than that of the parent strain. This method is efficient, cost-effective and provides wider application in screening for various surfactants.

## Introduction

Surfactin is an important biosurfactant, not only for its superior surface activity, but also its very promising antibacterial, antifungal, antimycoplasmic and antitumor activities [1], [2], [3]. Therefore, it has broad application prospects in the pharmaceutical industry. However, the high cost and low yield involved in surfactin production hamper its applications [4]. Thus, strategies for high-throughput strain screening and cost-effective production of surfactin are of urgent demand.

Surfactin belongs to a group of cyclic lipopeptides composed of polypeptide chain with seven D-/L-amino acids and a β-hydroxyl fatty acid chain [5]. Due to different length of the fatty acid chain, surfactin mainly contains three components: C_13_ surfactin, C_14_ surfactin and C_15_ surfactin, of which C_15_ surfactin has the highest activity including (a) surface activity [6], [7]; (b) hemolytic activity [8], [9]; and (c) antifungal activity [10]. Currently, the production of surfactin and C_15_ surfactin is at low level, which demands high-yield producing strains through strain improvement such as mutagenesis.

Traditional mutation is still an attractive approach for strain improvement. A successful search for high-yielding strains depends largely on the total number of mutants that can be screened after mutagenesis. However, an effective method is needed to find high-yield mutants. Previous studies have compared various screening methods [11], [12]. Some commonly used methods for analyzing biosurfactant production are blood agar [13], drop collapse [14] and tensiometric evaluation [15]. However, when many strains need to be assessed for surfactant production, none of them is practically feasible for high-throughput screening. Recently, there is a new high-throughput analyzing method named atomized oil assay, but its lowest detection limit is 250 mg/L [16], making it difficult to screen high-yielding mutants starting from low-yield parent strain.

Polydiacetylene (PDA) is a class of conjugated polymers that are developed as biosensors for surfactant owing to their unique color change from blue to red [17]. Recently, PDA has attracted more attentions, because it can be prepared in many forms, such as thin films in solid [18] or vesicles in water [19]. So far, PDAs with different modifications are widely used to detect cationic surfactants [20], [21] and ionic surfactants [22]. The detection signal of PDA sensors is usually the colorimetric response (CR%) that shows the relative change ratio of the blue form PDA and red form PDA. As reported, CR% of PDA sensors has been commonly used as a semi-quantitative method for many products; within the detection range, the larger the CR% value is, the higher the product concentration is [23]. However, PDA vesicles have never been used as sensors for detecting surfactin in fermentation broth, i.e. the effects of components and pH of fermentation broth on PDA vesicles have never been investigated, although microbial fermentation is the most attractive and feasible way to produce surfactin.

Here we present the development of a high-throughput screen method for surfactin producers based on the blue to red color change of PDA vesicles. With this method developed and validated, we found a few very promising high-yield surfactin producing strains after screening tens of thousands of mutants, which in consequence, will enable the cost-effective production of surfactin for its wide application.

## Materials and Methods

### Synthesis of PDA vesicles

The synthesis of PDA vesicles was done as previously described [24]. In brief, PDA vesicles were prepared by dissolving10, 12-pentacosadiynoic acid (PCDA) (97%, Sigma) in chloroform and rotary evaporated at 40°C. HEPES buffer (20 mmol/L, pH 7.4) was added to PCDA at a final concentration of 1 mmol/L and filtered through 0.45 µm filters. The sample was then sonicated at 70°C, 600 w for 15 min and then cooled at 4°C overnight to form PCDA vesicles. The PDA vesicles formed after irradiating the PCDA vesicles at wavelength of 254 nm for 3 min. The PCDA vesicles were stored at 4°C, and PDA vesicles were freshly synthesized from PCDA vesicles when needed.

### Colorimetric response (CR%) measurement

As sensor, PDA vesicles have blue to red color change when response occurs; and the degree of color change could be characterized by colorimetric response (CR%), which can be calculated according to the following equation [24]: 
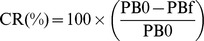
where PB is *A*_blue_/(*A*_blue_ + *A*_red_); *A*_blue_ is the absorbance at 650 nm; *A*_red_ is the absorbance at 540 m; PB_0_ and PB_f_ are values calculated before and after color change, respectively. The experiments were done with at least three replicates.

### Effect of fermentation broth on PDA vesicles

To study the effect of medium on PDA vesicles, 150 µL origin PDA vesicles, 150 µL deionized water, 150 µL blank Luria-Bertani (LB) medium and 150 µL LB medium containing 150 mg/L surfactin were mixed with 75 µL PDA vesicles, separately. The mixtures were keeping at 25°C for 10 min, and the CR% values of the mixtures were calculated.

To detect the effect of pH on PDA vesicles, a given surfactin concentration of 250 mg/L was selected, and the pH of the solution was adjusted by NaOH (1 M) or HCl (1 M) to 3, 4, 5, 6, 7, 8 and 9. In order to explore if the PDA vesicles were available for the fermentation system, the pH range from 6.0 to 8.2 with 0.2 gradients was assessed. The reactions were operated with 150 µL LB medium containing 250 mg/L surfactin and 75 µL PDA vesicles at 25°C for 10 min, then the CR% values were calculated.

Reaction ratio was an important factor to the PDA vesicles based screening method, the ratio between PDA vesicles and fermentation supernatant was selected at 3∶1, 2∶1, 1∶1, 1∶2 and 1∶3; and 9 concentrations of surfactin at 50, 100, 150, 200, 250, 300, 350, 400 and 450 mg/L were chosen in every ratio. The pH of LB medium containing surfactin was adjusted to 7.4, then the reaction was done at 25°C for 10 min, and the CR% values were calculated.

### Microorganisms and mutation

Surfactin-producing strain *Bacillus subtilis* 723 isolated from petroleum-contaminated soil from Shengli Oil Field, China, was selected as the parent strain. Atmospheric and room temperature plasma (ARTP) mutation method was applied for the mutation of *B.subtilis* [25]. Briefly, 10 µL of the culture (OD_600_ = 2.0) was dipped onto a sterilized stainless steel plate (4.0 mm in diameter) and dried in sterile air for a few minutes. The metal plate with the bacterial cells was then treated by the helium plasma jet for 150 s. The apparatus was operated at the helium gas flow rate of QHe = 10.0 slpm (standard liters per minute) and RF power input of 100 W. After treatment of the bacterial samples, the metal plate was washed with 990 µL of sterilized water, and diluted to a series from 10^-5^ to 10^-7^. Then, 200 µL of the diluent was spread onto the LB agar plates (5 g/L yeast extract, 10 g/L tryptone, 10 g/L NaCl, 20 g/L agar). After cultivation at 37°C overnight, individual colonies were selected, and then inoculated into MicroFlask by Duetz (24 square deep well plates, Applikon Biotechnology, Schiedam, Netherlands) with 2 mL LB medium and incubated aerobically at 37°C and 200 rpm for 24 h. Then 150 µL fermentation supernatant was reacted with 75 µL PDA vesicles at 25°C for 10 min, and the CR% values were calculated.

### Subcultures of mutant strains

The genetic stability of the mutants for surfactin production was evaluated by the serial subcultivation. After incubating the target mutant on LB agar medium, a colony was transferred into a 250 mL flask containing 30 mL fresh LB liquid medium and cultivated for 12 h (the first subculture). The culture was then used to inoculating 30 mL fresh LB liquid medium in a 250 mL flask and cultivated for 12 h (the second subculture). The same subculture procedure was repeated for 10 times. The surfactin production was checked in the 250 mL flask in every subculture.

### Extraction of surfactin

The isolation and extraction of surfactins were done by acid centrifugation and organic solvent extraction as reported elsewhere [26] with minor modifications. Specifically, high-yielding strains were cultured in LB medium aerobically at 37°C and 200 rpm for 24 h. Then, the culture broth was centrifuged at 8000×*g* at 4°C for 10 min to remove the cells from the solution, and 6 mol/L HCl was added to the supernatant until pH 2.0, refrigerated at 4°C overnight. After precipitation, the pellets were neutralized and extracted with methanol for three times.

### Analytical methods

The methanol extraction of surfactin was analyzed using an HPLC system (Dionex P680 Series, UT) equipped with an Amethyst C_18_-P column (5 µm, 120 Å, 4.6×250 mm). The mobile phase consisted of 90% methanol and 10% water (0.05% trifluoroacetic acid). After loaded with 20 µL filtered methanol extract, column was eluted at a flow rate of 0.8 mL/min at 30°C. The elution was monitored by the UV absorbance at 214 nm. The product types and relative content of surfactin were analyzed by LC-ESI-MS [27].

### Performance comparison of parent strain and high-yielding mutant

Parent strain and high-yielding mutant were inoculated into 250 mL flasks containing 30 mL fresh LB liquid medium and incubated aerobically at 37°C and 200 rpm for 12 h. Then 2% of inoculums were transferred to 250 mL Erlenmeyer flasks containing 50 mL LB fermentation medium and incubated aerobically at 37°C and 200 rpm for 12, 24, 36 and 48 h respectively. After fermentation, OD_600 nm_ and pH of broth were detected. Then the yield of surfactin and the proportion of surfactin variants (C_13_, C_14_ and C_15_ surfactin) were analyzed by HPLC after centrifuging and acid precipitating the culture broth.

## Results

### Effect of fermentation broth on PDA vesicles

**Figure 1:**
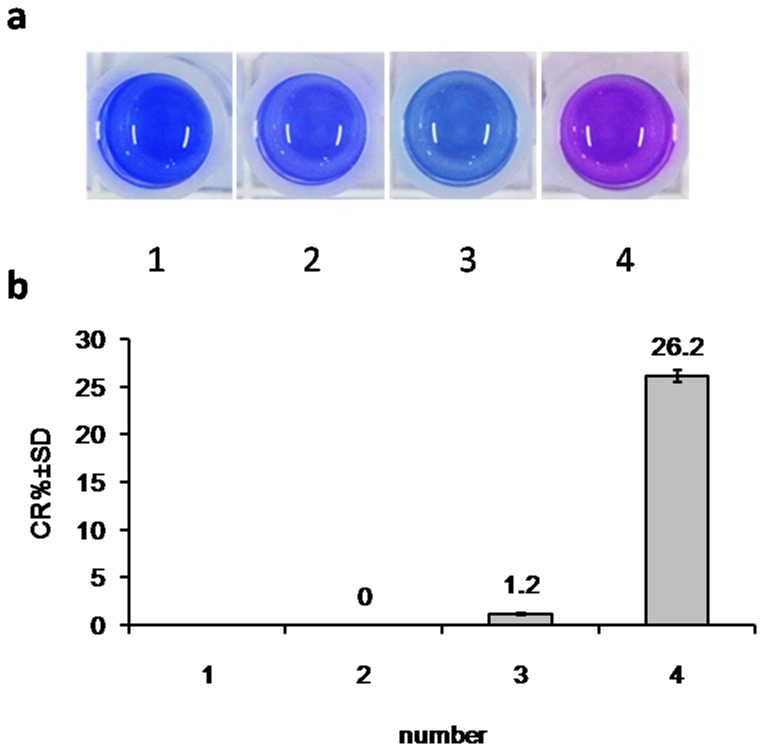
Effect of blank LB medium on colorimetric response. (a): the color of all mixtures; (b): the CR% values of samples. (1) was 225 µL original PDA vesicles; (2) was the mixture of 150 µL deionized water and 75 µL PDA vesicles; (3) was the mixture of 150 µL blank LB medium and 75 µL PDA vesicles ; (4) was the mixture of 150 µL LB medium containing 150 mg/L surfactin and 75 µL PDA vesicles.

As shown in Figure 1, blank LB medium had little interference on PDA vesicles and the CR% value. The higher the concentration of surfactin was, the smaller the effect of LB medium had.

**Figure 2:**
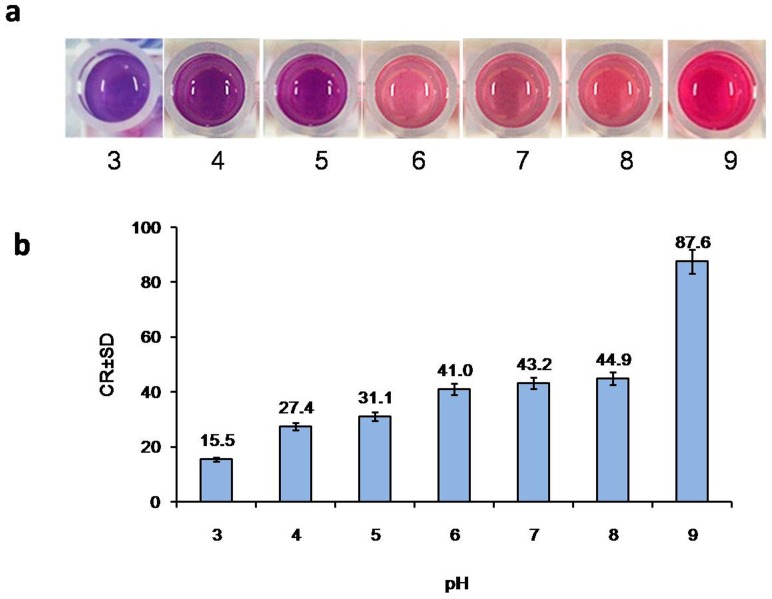
Effect of pH on colorimetric response. (a): the color of all mixtures; (b) the CR% values of samples. When detected the CR% values, the reaction system contained 150 µL blank LB medium with 250 mg/L surfactin and 75 µL PDA vesicles. The pH of reaction systems were adjusted with 1 M HCl or 1 M NaOH.

**Figure 3:**
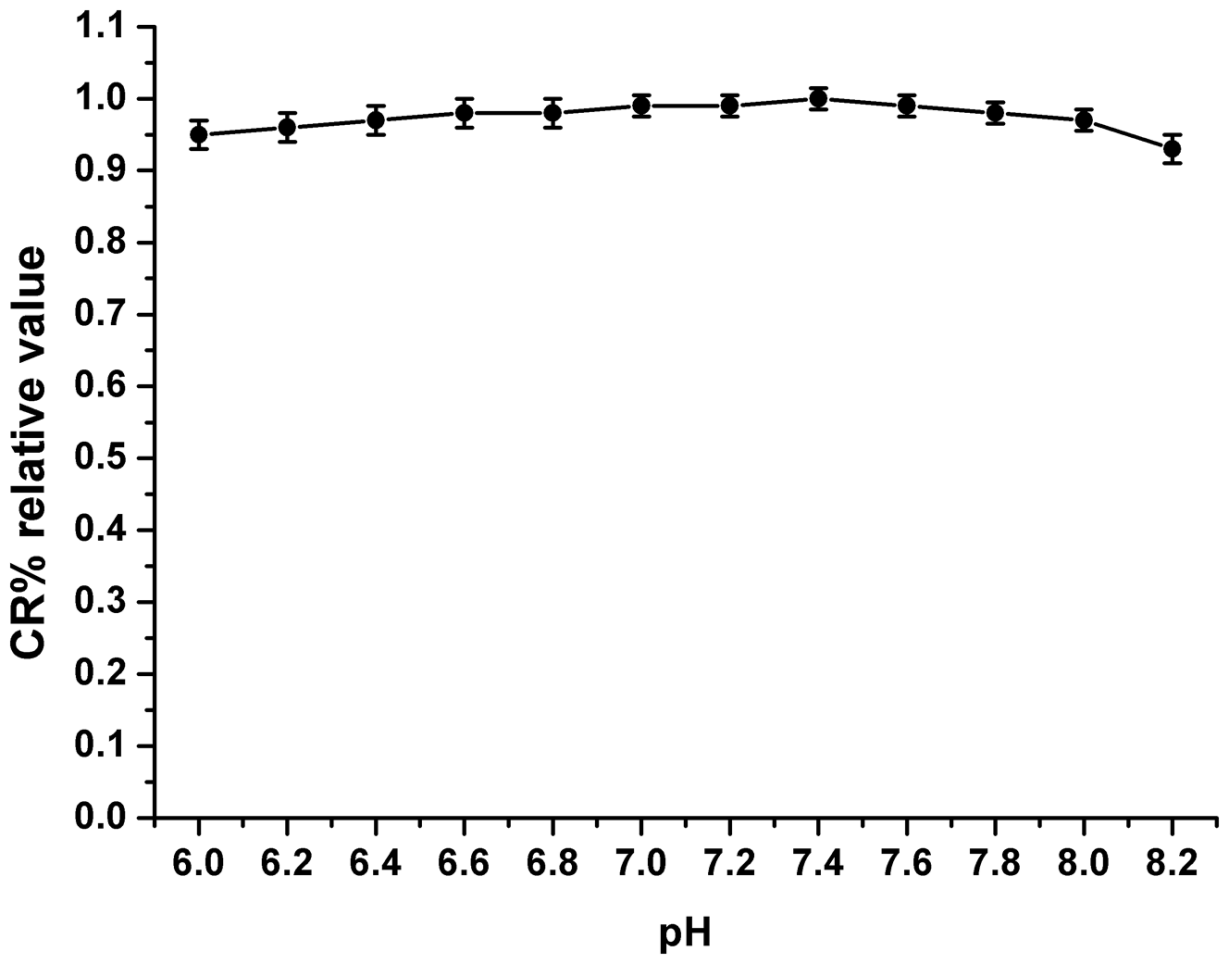
Effect of pH on colorimetric response. The reaction system was mixture of 150 µL LB medium containing 250 mg/L surfactin and 75 µL PDA vesicles. The pH of mixture was adjusted from 6.0 to 8.2 with 1 M HCl or 1 M NaOH. The reaction was kept at 25°C for 10 min. The CR% value of pH 7.4 reaction system was considered as 100%.

At a constant surfactin concentration (250 mg/L), PDA vesicles had a different color response at pH ranging from 3.0 to 9.0. As shown in Figure 2, the CR% values changed from 15.5 to 87.6%, which means pH had a significant effect on the color response of PDA vesicles. However, during the fermentation, the pH of broth always ranged from 6.0 to 8.0. Then, we narrowed the range of pH from 6.0 to 8.2 with a gradient of 0.2. As shown in Figure 3, pH ranging from 6.4 to 8.0 had no significant effect on CR% values, while exceeded this range was not the case.

**Figure 4:**
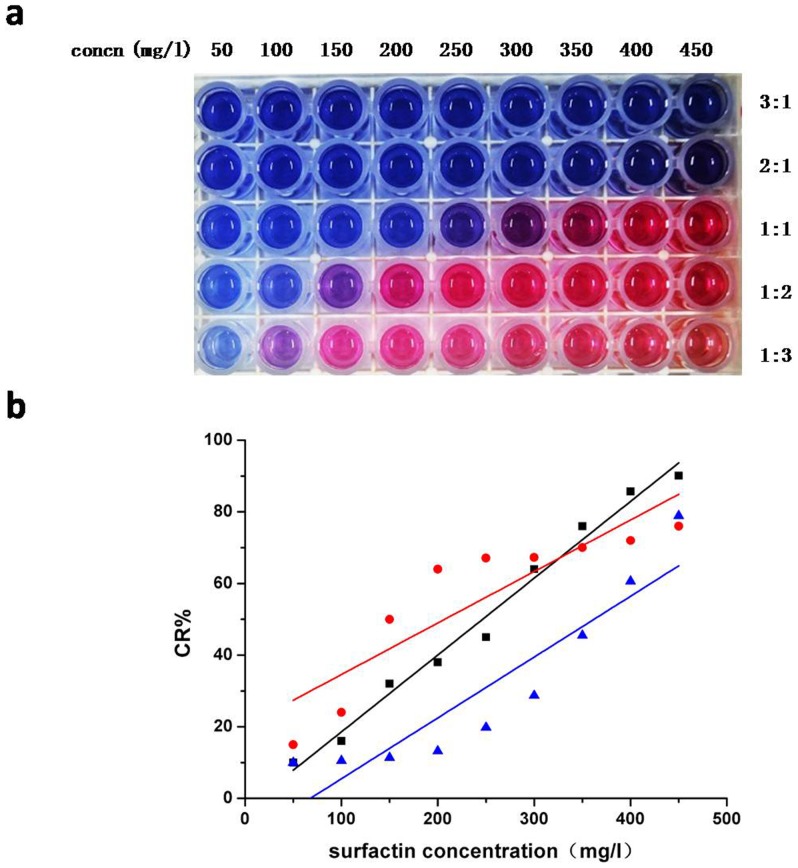
The optimization of reaction ratio. PDA vesicles were mixed with LB medium containing 50, 100, 150, 200, 250, 300, 350,400 and 450/L surfactin respectively at ratios of 3∶1, 2∶1, 1∶1, 1∶2 and 1∶3, then kept at 25°C for 10 min. (a): The color response of PDA vesicles. (b): The correlation between CR% and surfactin concentration at ratio of 1∶1 (Blue), 1∶2 (Black) and 1∶3 (Red). The R^2^ values for the linear regressions were 0.8264, 0.9862 and 0.8938 respectively. All the experiments were carried out in triplicate, and values are the average of three independent determinations.

The optimization of reaction system mainly focused on the ratio of PDA vesicles and different concentrations of surfactin. As shown in Figure 4 (a), when the ratio at 3∶1 and 2∶1, the reaction system had no significant color change. When the ratio was at 1∶1, 1∶2 and 1∶3, the lowest surfactin concentrations for the visible color change were 250, 150 and 100 mg/L, respectively. The curve fitting between surfactin concentration and CR% values was shown in Figure 4 (b), and the best linear regressions appeared at ratio of 1∶2.

### Selection of surfactin high-yielding strains

**Figure 5:**
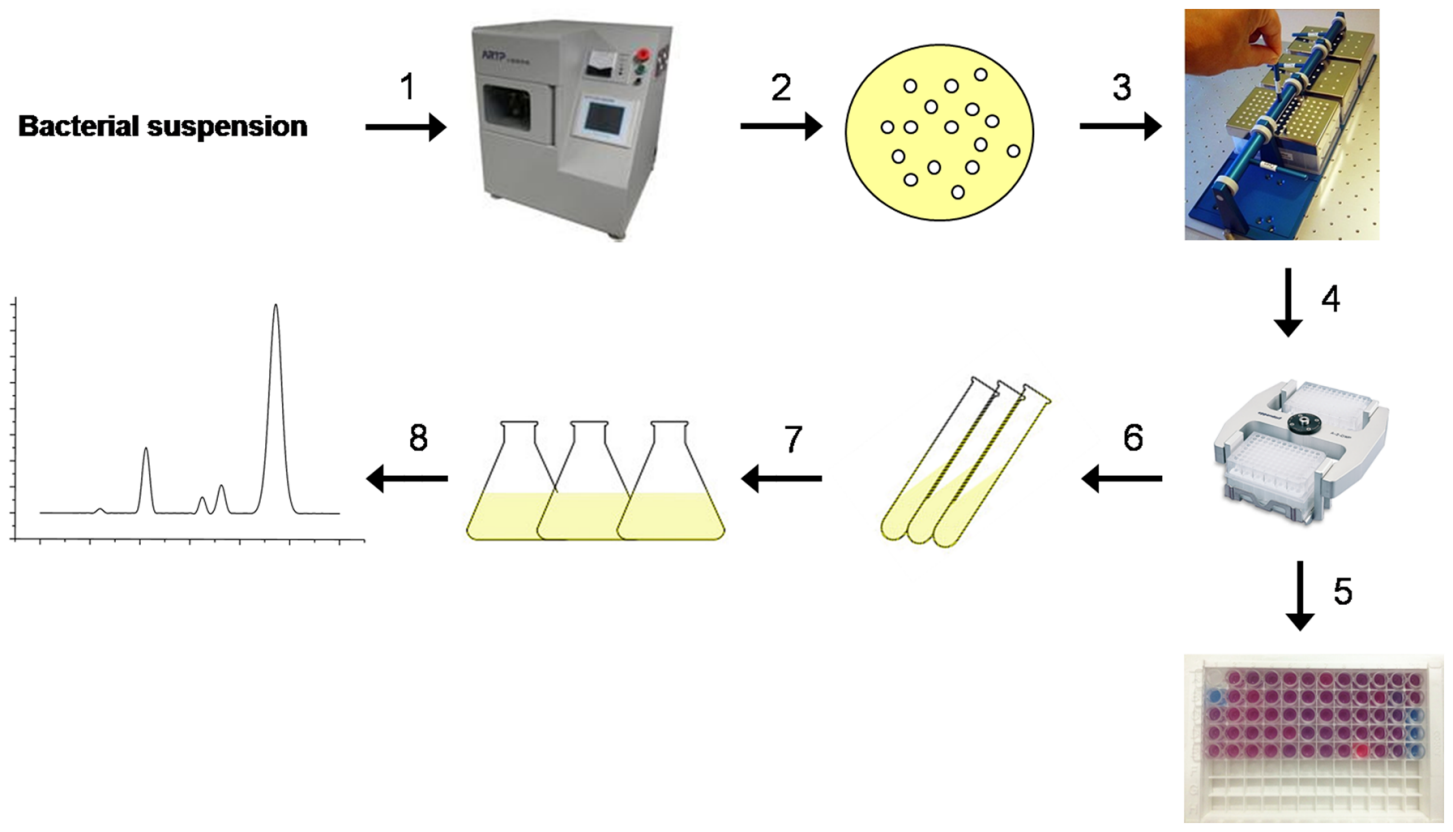
Strategy for screening surfactin producers. (Step1) Bacterial suspension of *B. sbtilis* 723 is mutated by ARTP; (Step 2) Mutants after gradient dilution are plated on LB agar at 37°C until clear individual colonies are observed (12 h); (Step 3) Select individual colonies to MicroFlask by Duetz and incubate aerobically at 37°C, 200 rpm for 24 h; (Step 4) The MicroFlask by Duetz with fermentation broths are centrifuged at 8000×*g* for 10 min; (Step 5) 150 µL supernatant and 75 µL PDA vesicles are reacted at 25°C for 10 min, and the CR% values are calculated; (Step 6) The strains with CR% more than 26% are preserved; (Step 7) The high-yielding mutants are cultivated under conditions appropriate for surfactin production (LB medium, 37°C), and (Step 8) The cell-free culture supernatant is collected and analyzed by HPLC to quantify the surfactin content in the medium.

After the above optimization processes of key operation conditions, the high throughput screening (HTS) procedure with PDA vesicles was successfully developed, and used to screen high surfactin-producing mutants (schematically shown in Figure 5). All mutants with CR% values above 26% (surfactin titer 150 mg/L) were selected as the positive candidates and preserved, and subsequently used as parent strains for further mutation to obtain higher yield of surfactin.

**Figure 6:**
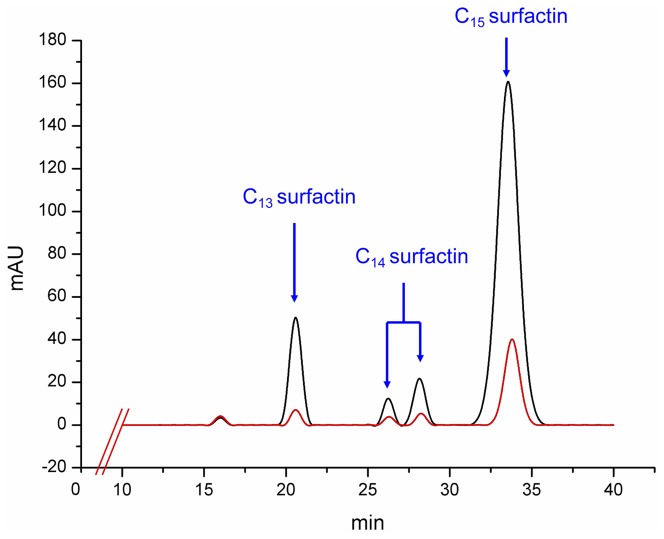
HPLC profile of surfactin produced by parent strain and mutant BS-37. Parent strain *B. subtilis* 723 and mutant BS-37 were cultured in 250 mL Erlenmeyer flask containing 50 mL LB medium aerobically at 37°C and 200 rpm for 24 h. The products were extracted by acid centrifugation and methanol extraction. The elution was monitored at 214 nm at a flow rate of 0.8 mL/min. The red line and the black line represent the HPLC profiles of surfactins produced by *B. subtilis* 723 and BS-37, respectively.

A total of 27000 separate colonies appeared in LB medium after multiple mutations with atmospheric and room temperature plasma. Of these, 26730 colonies grew well in MicroFlask by Duetz, and those with poor or no growth were discarded. By running the HTS procedure, 37 mutants were judged as promising candidates of high-yielding strains. We numbered these mutants from BS-1 to BS-37 according to the chronological order when obtained. The CR% values and the yields of those strains were detected (Table 1). From Table 1, when in a narrow range of concentration (a few milligrams), the CR% values had a little fluctuation, but when widened the range of concentration (more than 10 milligrams), the CR% values and the level of concentration had a good correlation. After HPLC quantitation, a high-yielding strain BS-37 was selected. The HPLC profile of parent strain *B. subtilis* 723 and mutant BS-37 was shown in Figure 6, the titer of *B. subtilis* 723 was 87.8 mg/L, whereas that of BS-37 was 473.6 mg/L, a 5.4- fold increase was obtained and the final titer of C_15_ surfactin was 353.1 mg/L.

**Table 1:** CR% values and titers of surfactin.

Number	CR% ± SD	Titer (mg/L) ± SD	Number	CR% ± SD	Titer (mg/L) ± SD
Parent strain	16.5±0.4	87.8±1.8	BS-2	41.3±1.5	219.7±2.7
BS-37	100.0±1.8	473.6±6.6	BS-7	41.1±0.7	215.8±3.6
BS-36	90.7±1.6	438.4±5.8	BS-26	40.1±0.8	211.2±4.4
BS-35	78.0±1.3	388.6±4.6	BS-6	40.2±0.9	210.1±4.9
BS-33	65.6±0.6	310.9±4.9	BS-19	39.7±2.0	208.3±4.0
BS-29	61.0±1.6	302.7±3.6	BS-22	38.5±0.6	204.0±3.9
BS-32	55.1±0.9	287.5±4.4	BS-13	37.3±1.1	196.7±3.4
BS-28	55.4±1.1	283.7±4.0	BS-1	36.4±1.5	192.0±2.9
BS-10	52.3±1.1	280.6±3.3	BS-11	35.2±1.3	188.8±3.4
BS-24	48.7±1.2	271.0±4.5	BS-14	34.2±1.5	186.6±3.9
BS-18	46.5±1.0	266.2±3.8	BS-21	32.4±1.1	184.6±3.9
BS-34	45.8±0.6	260.6±2.4	BS-9	32.6±1.0	180.6±2.0
BS-8	44.3±1.4	257.7±3.5	BS-16	32.7±1.5	177.9±4.3
BS-12	44.7±1.0	252.9±2.0	BS-20	31.1±1.2	174.5±3.5
BS-4	43.8±2.0	245.6±4.2	BS-23	31.2±0.8	171.7±4.4
BS-30	42.1±1.2	240.8±3.6	BS-15	30.3±0.9	166.4±3.7
BS-5	42.3±1.2	233.5±2.8	BS-17	30.1±1.1	165.8±1.9
BS-3	41.8±1.3	225.7±3.7	BS-25	28.5±0.8	160.7±3.3
BS-31	42.3±1.7	224.3±3.5	BS-27	26.6±0.6	158.3±2.1

The parent strain and 37 positive mutants were cultivated in 250 mL Erlenmeyer flask containing 50 mL LB medium aerobically at 37°C and 200 rpm for 24 h, and each strain was incubated in triple. For CR% test, 150 µL supernatant of culture and 75 µL PDA vesicles were reacted in 96-well plate at 25°C for 10 min.

### Stability of high-yielding strain

Stability of the mutant was evaluated by serial subcultures. After10 subcultures of the high-yielding strain BS-37, surfactin production stability of the mutant was maintained (data not shown here), suggesting that mutant BS-37 with a high genetic stability.

### Characterization of surfactin in BS-37

**Figure 7:**
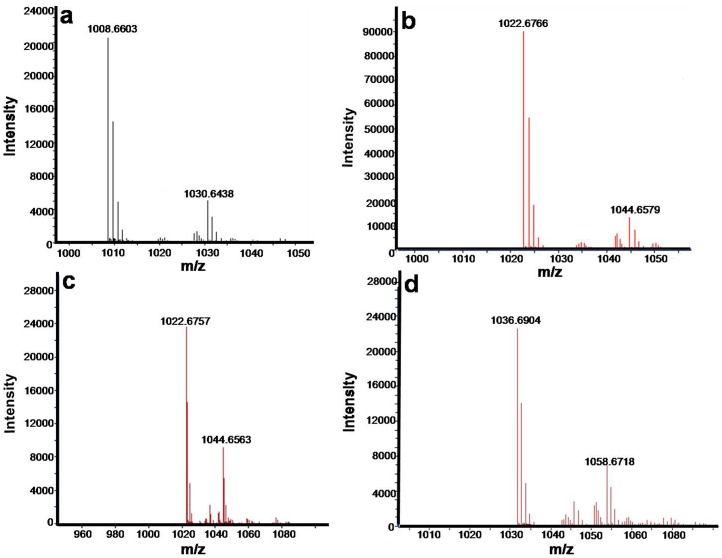
ESI spectra of the surfactin extract of BS-37. BS-37 was cultured in 250 mL Erlenmeyer flask containing 50 mL LB medium aerobically at 37°C and 200 rpm for 24 h. The product was extracted by acid centrifugation and methanol extraction. The MS conditions were: capillary temperature 250°C, source voltage 4.0 kV, source current 80.0 µA, and capillary voltage 7.0 V, in positive mode.

As shown in Figure 5, the molecular mass of the purified compounds was measured using ESI-MS spectrometry, giving main peaks at m/z = 1008, 1022, 1022, 1036 ([M+H]^+^) and m/z = 1030, 1044, 1044, 1058 ([M+Na]^+^) (Figure 7), corresponding to the molecular mass of C_13_ surfactin, C_14_ surfactin (twice) and C_15_ surfactin, respectively. The relative content of C_13_ surfactin, C_14_ surfactin and C_15_ surfactin was13.60, 11.85 and 74.55%, respectively (Table 2), obtained by LC-MS analysis.

**Table 2:** LC/ESI-MS analysis of the composition and relative percentage of homologues in surfactin extract.

MW	surfactin type	Retention time(min)	Relative content (%)
1008	C_13_ surfactin	20.56	13.60
1022	C_14_ surfactin	26.25	4.24
1022	C_14_ surfactin	28.16	7.61
1036	C_15_ surfactin	33.55	74.55

### The performance difference between parent strain *B. subtilis* 723 and mutant BS-37

The fermentation performance including OD_600 nm_, pH, titer of surfactin and proportion of surfactin variants of *B. subtilis* 723 and BS-37 were shown in Table 3. The values of OD_600 nm_ in BS-37 had a significant decrease compared with that in *B. subtilis* 723, and the OD_600 nm_ value in BS-37 was dramatically reduced at 36 h. For both parent and mutant, there was no significant difference of pH of culture broth; the pH fluctuations were in the range of 6.8 to 7.6 during the fermentation process. The highest yields of surfactin in parent and mutant were occurred at 36 h, with titers of 143.1 mg/L and 611.3 mg/L, respectively. For both parent and mutant, the proportion of surfactin variants changed during the fermentation process with the decreasing of C_15_ and increasing of C_13_ and C_14_. However, the proportions of C_15_ in surfactin variants produced by mutant were much higher than those of parent.

**Table 3:** Performance of parent strain *B.subtilis* 723 and mutant BS-37.

time	OD_600 nm_		pH		Titers of surfactin (mg/L)		Proportions of variants (%)^a^	
	parent	mutant	parent	mutant	parent	mutant	parent	mutant
12 h	5.5	3.3	6.8	6.9	74.4	321.1	25∶0∶75	9∶0∶91
24 h	9.1	5.2	7.2	7.3	87.8	473.6	30∶12∶58	14∶10∶76
36 h	8.4	4.7	7.4	7.5	143.1	611.3	53∶15∶32	33∶16∶51
48 h	7.3	5.1	7.5	7.6	84.8	514.0	55∶12∶33	36∶10∶54

a The proportion of C_13_ surfactin, C_14_ surfactin and C_15_ surfactin.

## Discussion

### Applicability and validation of PDA vesicles as sensors for biosurfactant

PDA vesicles response to a variety of external stimuli such as temperature, pH, and molecular recognition [28], [29]. Screening for microbial products, in our study surfactin, is carried out in synthetic media that are normally composed of complex components including carbohydrates, proteins and minerals. Moreover, different microbial culture requires different optimal pH value. In our study, LB medium had no significant effect on PDA vesicles and CR%, confirming that LB medium is suitable to screen for surfactin producing strains with this assay (Figure 1). Our results also indicate that neutral pH range is suitable for PDA vesicle (Figure 2), agreeing with previous results that both acid and alkali conditions can cause the color transition [30], [31]. Although medium pH might fluctuate during the fermentation process, the fluctuation range within pH 6.4 to 8.0 had little effect on the chromogenic reaction (Figure 3). Our study confirms that PDA vesicles-based method was suitable for the medium used within the fermentation pH range. The applicability of the PDA vesicles-based screening method was further validated by HPLC analysis. By comparing the CR% values with HPLC quantitation results of 37 high-yielding strains (Table 1), the screening method is proved feasible and reliable.

### Comparison of PDA vesicles-based high-throughput screening with other methods

The lack of high-throughput strain improvement remains a bottleneck in cost-effective production although microbial production of surfactin has been extensively studied and reviewed in recent years [6], [7], [8], [9], [10]. Compared to other screening methods, our study confirms the advantages of PDA vesicles-based screening method (Table 4). Compared with blood agar method, PDA vesicles is more cost-effective. For example, to screen 10,000 mutants, it needs only 0.3 g 10, 12- pentacosadiynoic acid (PCDA) (97%, Sigma) for preparing PDA vesicles, which costs no more than 10.5 US dollars. If using blood agar method, it needs 500-1000 mL sterile defidrinated sheep blood (5% blood in blood agar), which costs 75.0-150.0 US dollars. Moreover, the size of transparent circles in blood method affected by hemolysins produced in bacteria, and not all biosurfactants have a hemolytic activity [32], so it is easy to cause analysis errors. In contrast to drop collapse screening method, PDA vesicles method is more efficient to achieve the semi-quantification, and the sensitivity is higher [16]. Although the Atomized oil method is a high-throughput with semi-quantitative advantage, the experimental phenomena is indirectly visual [16] and the sensitivity is low in detecting surfactin. The latter, in practice, can cause false negative screening results when the starting strain or parent strain has low surfactin yield whereas its mutants might have unexpected potentials.

**Table:** **Table 4.** Comparison of different screening methods.

Method	Detection cost^a^ (US dollar)	Limit of detection^b^ (g/L)	Quantitative analysis	Analysis speed
PDA vesicles	10.4	0.05	++	++
blood agar	75.0–150.0^c^	-	+	+++
drop collapse [16]	-	15.00	+	++
Atomized oil [16]	-	0.25	++	++

+++  =  highest efficacy.

a The detection cost for 10000 mutants.

b These values are the lowest titers of surfactin that still occurred visual detection by the respective assays.

c To compare the size of transparent circles in blood agar, the average colonies on one agar plate should be limited in 10–20 (depends on the titers of surfactin). So it needs 500–1000 pieces of blood agar plates containing 10–20 L culture medium. It needs 500–1000 mL defibrinated sheep blood (5% blood in blood agar), which costs 75–150 US dollars.

### Achieving a high-yield strain through high-throughput screening

By the PDA-based high-throughput screening method, a high-yielding strain BS-37 was obtained from 27,000 mutants. Its surfactin titer in 250 mL Erlenmeyer flask containing 50 mL LB medium was 473.6 mg/L after 24 h cultivation, which was a relatively high yield [10], [32], and it could inherit stably after more than 10 generations. By LC-ESI-MS analysis, we obtained the quasi-molecular ions, comparing to the standard, the product was identified as surfactin, and the four homologues were C_13_ surfactin, C_14_ surfactin (twice) and C_15_ surfactin.

### Potentials of applying PDA vesicles-based screening method

The PDA structure, which is conjugated through 1, 4-addition of diacetylenic monomers upon UV irradiation at 254 nm, appears visible blue. It yields a significant blue-to-red chromatic change in response to a variety of external stimuli, leading to the development of a variety of PDA-based chemosensors, especially for the colorimetric detection of biologically important target molecules such as DNAs [33], viruses [34] and proteins [35]. Our results confirm that the developed PDA-based screening for surfactin is feasible, sensitive and reliable. In addition, this assay can be broadened for many other surfactants as PDA in different forms were used to detect the ionic surfactant [20], [21], [22]. In our study, PDA-based screening method was used to determine surfactin as the model biosurfactant; such a screen method may be readily applied to screen many other biosurfactant, including glycolipids, phospholipids, lipoproteins or lipopeptides, polymeric compounds, mycolic acids and lipopoly saccharides [36] to which surfactin, one of the lipopeptides, belongs to.

## Conclusions

We developed an applicable, sensitive and reliable high-throughput screening assay for biosurfactants. Using this method, we obtained in a cost-effective way a very promising high-yield strain of *Bacillus subtilis* with genetic stability and high product concentration in fermentation broth. Moreover, the principle of PDA vesicles-based screening method can be readily applied to other applications, such as screening for glycolipids, phospholipids, lipoproteins or lipopeptides, polymeric compounds, mycolic acids and lipopoly saccharides.
